# Interaction of the pre- and postnatal environment in the maternal immune activation model

**DOI:** 10.1007/s44192-023-00042-5

**Published:** 2023-08-22

**Authors:** Anna Gundacker, Laura Cuenca Rico, Peter Stoehrmann, Katharina E. Tillmann, Ulrike Weber-Stadlbauer, Daniela D. Pollak

**Affiliations:** 1 Department of Neurophysiology and Neuropharmacology, Center for Physiology and Pharmacology, Medical University of Vienna, Schwarzspanierstrasse, 17, 1090 Vienna, Austria; 2 Institute of Pharmacology and Toxicology, University of Zurich-Vetsuisse, Zurich, Switzerland; 3 Neuroscience Center Zurich, University of Zurich and ETH, Winterthurerstrasse 190, 8057 Zurich, Switzerland

## Abstract

Adverse influences during pregnancy are associated with a range of unfavorable outcomes for the developing offspring. Maternal psychosocial stress, exposure to infections and nutritional imbalances are known risk factors for neurodevelopmental derangements and according psychiatric and neurological manifestations later in offspring life. In this context, the maternal immune activation (MIA) model has been extensively used in preclinical research to study how stimulation of the maternal immune system during gestation derails the tightly coordinated sequence of fetal neurodevelopment. The ensuing consequence of MIA for offspring brain structure and function are majorly manifested in behavioral and cognitive abnormalities, phenotypically presenting during the periods of adolescence and adulthood. These observations have been interpreted within the framework of the “double-hit-hypothesis” suggesting that an elevated risk for neurodevelopmental disorders results from an individual being subjected to two adverse environmental influences at distinct periods of life, jointly leading to the emergence of pathology. The early postnatal period, during which the caregiving parent is the major determinant of the newborn´s environment, constitutes a window of vulnerability to external stimuli. Considering that MIA not only affects the developing fetus, but also impinges on the mother´s brain, which is in a state of heightened malleability during pregnancy, the impact of MIA on maternal brain function and behavior postpartum may importantly contribute to the detrimental consequences for her progeny. Here we review current information on the interaction between the prenatal and postnatal maternal environments in the modulation of offspring development and their relevance for the pathophysiology of the MIA model.

## Introduction

The internal and external environment of the mother during pregnancy is critically important for the development of the mammalian fetus, determining its path to health and disease. The intrauterine period encompasses extensive developmental processes, from the implantation of the zygote, the differentiation of precursor cells and organogenesis, to the final functional organ development of an expansive growth of the fetus [1, 2]. This unique transformation from fertilized egg to new intact organism most impressively includes the generation and development of the nervous system, which starts with the formation of the neural tube, the establishment of the main structures of the brain and involves the coordinated differentiation and migration of neural cells as prerequisite for the initial wiring with other neurons, finally leading to the construction of the first neural networks [1, 2]. However, a fundamental part of brain development occurs after birth, with an expeditious expansion of neuropil and glial cells accompanying the remodeling and experience-dependent refinement of existing neural circuits and the creation of new connections [3–5].

In order to accommodate the developing fetus and responding to its changing and growing demands throughout gestation, the pregnant female also has to undergo significant anatomical and physiological changes [6–8]. These dynamic processes, enabling a healthy pregnancy and providing the conditions for optimal development of the offspring, also serve to prepare the mother for the requirements of the post-partum period. Most importantly, the period of matrescence involves a high level of neuroplastic adaptions in the female brain, which accompany her changing throughout pregnancy to motherhood [9]. The modulatory mechanisms of structural and functional neural rearrangements enable the full range of care and nurturing behavior a mother displays towards her newborn immediately after birth, which are pivotal for their survival and ability to thrive.

This enormous capacity for brain development, dynamic adaptation and refinement of present neural architecture and functional circuitry, demand a high degree of plasticity [10]. The underlying malleability comes at the price of heightened sensitivity to external influences and consequently, presents a window of vulnerability to environmental adversities, of both mother and child [2]. As such, infections of the mother during pregnancy leading to maternal immune activation (MIA) are known to hamper the integrity and/ or quality of fetal development [2, 11–13] and are associated with a range of negative consequences for offspring social, emotional and cognitive functions [11, 14–16]. Direct mother-placental-fetal transmission exists for some infections agents, such as the Zika virus, *Toxoplasma gondii*, rubella virus, cytomegalovirus and the herpes simplex virus [17–21]. However, activation of the maternal immune system is common to all infections leading to a cascade of pathophysiological events which finally disrupt the tightly controlled balance between the maternal and fetal compartments. To explore the underlying principles, different MIA animal models exist which have jointly contributed to a robust body of knowledge demonstrating that activation of the maternal immune system is a shared pathophysiological mechanism that mediates the consequences of several infectious and non-infectious conditions during pregnancy on offspring brain development.

The present review will assess how infection-mediated MIA impacts the maternal–fetal interface during pregnancy and the perinatal maternal physiological adaptations, both of which can, presumably independently and jointly, exert life-long and even generation-spanning effects on offspring health.

### Fetal programming

The fine-tuned series of spatially and temporally constrained events required for optimal fetal development are intrinsically dependent on a balanced intrauterine environment that ensures the adequate supply of macro- and micronutrients, oxygen, hormones, and other regulators of guidance and growth. The fetus is entirely reliant on the maternal organism and communicatory mechanism that enable the most beneficial allocation of resources, matching its demand at a given time point in development. In most mammals the placenta is the main site for maternal–fetal exchange. The placenta not only constitutes a passive interface and transport system, but it is also actively engaged in dynamically modulating supply in response to changing maternal and fetal signals [22]. During evolution, this mechanism has evolved to maintain equilibrium between fetal growth and maternal survival, especially under conditions of scarcity.

As offspring largely grow close to their parents, this also prepares the fetus for the external conditions, which it will face upon birth. Consequently, the fetal organism adapts its blueprint of developmental processes in response to the changes communicated via the placenta, which mirror the environment its mother is confronted with [2].

This process of “fetal programming” was first described by David Barker in the context of inappropriate nutritional support in utero and the “programming” of offspring metabolic characteristics [23]. The corresponding “*fetal origins hypothesis*” postulates that this developmental programming in response to adverse influences during the intrauterine period determines the risk for the development of diseases later in the life. Although originally focused on the effects of inadequate nutrition in the prenatal period and its association with metabolic derangements, other early life environmental influences have since then been shown to have persistent health effects [24–30]. Epidemiological studies and work in animal models have revealed a role for maternal alcohol use, smoking, stress exposure and infection and an individual’s risk for a broad spectrum of pathologies in adult life, which importantly include neurological and psychiatric illnesses [31–42].

Within the framework of the “*fetal origins hypothesis*”, fetal programming can induce a variety of effects, ranging from (epigenetic) modulation of gene expression or long-term hormonal changes, to adjustments in metabolism, as well as to changes in basic biological functions like receptor cell density or sensitivity [43].

These molecular, cellular, structural and functional changes in the offspring organism are characterized by their persistency and long latency until the manifestation of disease, often triggered or “unmasked” by a second stimulus later in life [44–46].

In the following section we will focus on gestational infection and the maternal immune activation (MIA) model to exemplify how the impact of the interaction between prenatal adversities and the postnatal environment can shape neurodevelopment to contribute to the emergence of neuropsychiatric disorders.

### Neurodevelopmental consequences of maternal immune activation

Of the many potential adverse events during pregnancy, infections of the mother were among the first for which adverse effects on fetal neurodevelopment manifesting in alterations of offspring brain function have been demonstrated. Evidence is spanning from rubella embryopathy [13, 47] and congenital infections with *Toxoplasma gondii* [48–51], to the seminal epidemiological observations that after seasonal outbreaks or epidemics of rubella, influenza, measles, mumps and polio the number of cases of autism spectrum disorders (ASD) and schizophrenia (SZ) increased in the next generation [52–60]. Recently, large cohort studies have confirmed an increased risk of psychopathologic conditions in children with a history of infection during prenatal life [61]**.** In this context, the consequences of the COVID-19 pandemic for the incidence of neurodevelopmental disorders and the probability of psychiatric disorders in the offspring generation yet remain to be determined [62–66].

The establishment and successful application of valid and reliable animal models of MIA in the past 30 years have significantly advanced preclinical research into the neuropathological sequelae of gestational infection on offspring brain structure and function. There are excellent reviews available, contrasting and comparing the various existing MIA paradigms [67–76]. Herein, we will focus on the most widely used Poly(I:C) (Polyriboinosinic polyribocytidylic acid) model and current insights on potential interactions of the pre- and postnatal environments in shaping offspring neurophenotype.

Poly (I:C) is a double-stranded RNA (dsRNA) analog composed of homo-polymers of inosine and cytidine nucleotides. The immune system of mammals has evolved to recognize dsRNA through Toll Like Receptor 3 (TLR3). Consequently, binding of Poly (I:C) to TLR3 induces a cytokine-associated viral-like acute-phase response by activating the nuclear factor kappa-light-chain-enhancer of activated B cells (NK-kB) and Interferon (IFN) Regulatory Factor (IRF3) pathways [77, 78]. These signaling cascades increase the expression of pro-inflammatory cytokines that can induce cellular and molecular changes in different cell types, including neurons and glia [77]. Indeed, specific cytokines, such as Interleukin-6 (IL-6), interleukin-1ß (IL-1ß), interleukin-17A (IL-17A) [79–81] and Tumor Necrosis Factor-α (TNF α) have been shown to be necessary for the effect of Poly (I:C) MIA [77, 78, 82]. Importantly, TLR3 is also abundantly expressed in the placenta [77, 78] and Poly (I:C) can activate the placental immune response [83], suggesting a direct consequence of MIA at the maternal–fetal interface. The dire consequences of MIA on the intrauterine milieu in the fetal compartment are not exclusively meditated by the direct effects of proinflammatory cytokines, but also include a consequence of the concomitant restriction in nutritional and oxygen supply and an increase in reactive oxygen species [67, 78, 84–86].

Over the years it has become recognized that several procedural parameters importantly determine the outcome of Poly (I:C) MIA. These include the dosage and source of Poly (I:C) [66, 68, 74, 87, 88], the gestational time point of administration [11, 74, 87, 89–92], species and strain of the experimental animal [66, 76, 93–95] and its environment [15, 96–101] and these are comprehensively summarized and discussed elsewhere [46, 75, 76, 78, 99, 102–104].

The characteristics of the experimental design undoubtedly shape the response of the maternal system to the immune challenge hereby modulating its impact on the neurodevelopmental processes underlying offspring phenotype. However, the behavioral consequences of MIA for phenotypes related to neuropsychiatric disorders, including the anxiety disorder and depression, as well as autism spectrum disorders and schizophrenia have been repeatedly and robustly demonstrated across a wide range of experimental set-ups and animal species [16, 46, 74–76, 78]. As MIA can induce a wide spectrum of behavioral dysfunctions with face validity for distinct psychiatric entities in humans, MIA is not seen as a disease-specific risk factor, but rather as element derailing fetal neurodevelopment and enhancing the predisposition to psychopathology, possibly via as shared mechanisms [46, 76, 105–107]. Insights into the underlying mechanisms have substantially increased over the years and led to the identification of systemic, cellular, and molecular derangements used to explain the emotional, social, and cognitive disturbances of MIA offspring. The reader is referred to comprehensive recapitulations of the accumulated body of information on alterations in offspring neural composition, architecture, function and connectivity presented elsewhere [11, 16, 69, 74, 93, 96, 98, 101, 105, 107–117].

Here we would like to specifically point towards the role of epigenetic mechanisms, which are emerging as central regulators of long-term changes in gene expression in the MIA offspring brain. It is conceivable that the alterations in brain structure and function of MIA offspring, persisting beyond the early periods of intrauterine and postnatal brain development, are upheld by modifications of the epigenome driving life-long biased transcriptional programs. Moreover, epigenetic mechanisms are central to the physiological programming of brain development [69, 96, 100, 107, 118–120] and deregulations of such are implicated in a range of neuropsychiatric disorders, including those prominently related to gestational infection.

As such, the significance of disturbances of the neuroepigenome and its implications for the disruptions of neural differentiation, migration and circuit formation in the pathophysiology of schizophrenia is increasingly recognized [121–125]. Similarly, epigenetic marks in ASD patients are not only emerging as important diagnostic and prognostic markers but also offer insight into the etiological principles of ASD [126–128]. For depressive disorders, also strongly associated with maternal infection during pregnancy epidemiologically [61, 91, 93, 129–131], the importance of epigenetic principles in the pathomechanistic events leading to and/ or accompanying disease development has been long recognized [69, 99, 132–139].

With regards to preclinical MIA models, research of the last decade has provided convincing experimental evidence for an impact of early-life exposure to an immunogenic insult on the major epigenetic mechanisms and started uncovering how they can contribute to derailing the trajectories of neurodevelopment. Changes in DNA methylation patterns in the brains of MIA offspring were observed both globally [13, 70, 111, 132, 140, 141] and individually affecting promoter regions of genes with critical roles in neural functions [116, 120, 142]. Similarly, alterations in histone modifications were shown universally in different brain areas of MIA offspring [71, 107] and specifically uncovered in the regulatory regions of selected candidate genes [96, 143]. MIA also leads to persistent changes in gene expression through its effect on miRNAs and their dependent transcriptional networks [139, 144].

Jointly, these observations suggest MIA to directly interfere with the epigenetically determined program of gene expression and its control of neural development and brain function throughout life. In light of the influence of environmental stimuli on epigenetic mechanisms [145–148], the epigenome may also constitute the molecular interface at which the prenatal influence of an immunogenic stimulation converges with other elements of the postnatal environment. This interaction between MIA and other, additional adversities experienced later in offspring life, is of relevance within the framework of the “*double-hit*” hypothesis of neurodevelopmental disorders and may importantly determine the heterogeneity of phenotypes observed in the MIA progeny.

### Shaping offspring outcomes after MIA: the modulatory impact of the prenatal environment and the interaction with the postnatal environment

The effect of MIA can be influenced by a variety of factors, such as type and intensity of the immune stimulation, route of administration and gestational time point. It seems plausible, that other crucial factors in this context are variables of the maternal organism. These include the nutritional status of the mother, with the availability of macro- and micronutrients [86, 149–151], the maternal microbiome [75, 81, 152, 153], and stress exposure of the mother [37, 154–156], all of which can influence the MIA response. The nature of these, and possibly other factors, of the prenatal environment may contribute to the heterogeneity of phenotypes reported across different MIA studies. Indeed, while historically a large degree of variability in outcomes after an experimental manipulation was considered a “weakness” of the paradigm used, it is becoming more appreciated that the wide spectrum of responses obtained in MIA studies harbors a great potential for unraveling the mechanisms controlling the path to pathology, regulating disorder specificity and modulating susceptibility and resilience [75, 78, 111].

Additionally, considering the accepted role of gestational infection as risk factor predisposing to disease development, rather than categorically determining a preformed trajectory to a defined illness, the observed diversity of individual phenotypes may powerfully enhance the translational relevance of MIA models.

**Fig. 1:**
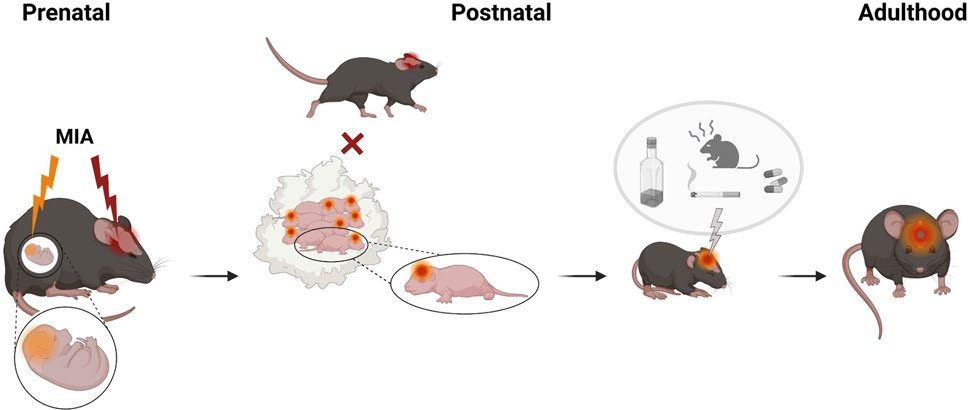
Interaction between the prenatal and postnatal environments shape for offspring brain and behavior outcomes. Maternal immune activation (MIA) directly disrupts neurodevelopment of the fetus in-utero, while at the same time impinging on the maternal brain during pregnancy. Disturbances of the neural rearrangements in the mother´s brain negatively affect postpartum maternal care behavior which can act as a second hit during postnatal development of the offspring. Additional adverse influences in offspring life, such as drug abuse or stress exposure during adolescence, may further determine an individual´s susceptibility and risk for a specific psychopathology

To date, most psychiatric disorders are recognized to be of multifactorial etiology, a consideration that has long been formulated as “*dual hit hypothesis*” in schizophrenia research [44, 157]. This theory postulates that exposure to a first insult, usually in the form of a genetic predisposition or an adverse environmental exposure prenatally may only induce subthreshold deviations of the physiological trajectories of brain development leading to a latent phenotype in a primed organism. A subsequent interaction with a second “hit” may then induce the manifestation of an apparent phenotype and actual emergence of the disease (Fig. 1.). Specific periods during life are known as “windows of vulnerability” during which environmental adversities can have powerful impacts. This is the result of a highly plastic brain, which undergoes fundamental neural rearrangements and dynamic restructuring requiring heightened malleability, which at the same time also allows for the substantial repercussions of external influences during these periods. As such, adolescence is viewed as a particularly sensitive phase of the mammalian brain development, where environmental impacts can interfere with the ongoing processes of dynamic rewiring [158, 159]. Here, an already “primed” brain is specifically vulnerable to the consequences of trauma, stress and drug abuse as second hits, finally governing the path to pathology [98, 148, 160–162]. As such, most of these factors have been studied in the context of gestational infection [163] and mechanistically explored in preclinical MIA models [98, 113, 164, 165].

### Postnatal care behavior and the maternal brain

Developmental psychology has long highlighted the early postpartum period as a phase of remarkable sensitivity when stimuli of the outside world have the capacity to shape the ongoing wiring of the brain in an incomparable manner. Famously postulated in the theory of “*imprinting*” by Konrad Lorenz, the consequences for behavior are considered to be largely irreversible [166].

In mammals, parental care behavior, mostly maternal care [167–170], constitutes the strongest external influence during early postnatal life and its critical importance for offspring physiological, psychological and social development is robustly well-established and robustly demonstrated [10, 171–183]. Displaced or dysfunctional parental care behavior can have severe and long lasting neuropsychiatric and medical consequences for the offspring; indeeed parental maltreatment, abuse, and neglect have been directly associated with irreversible cognitive alterations in children [175, 184–190].

In rodents, tactile stimulation by licking and grooming of pups is an important component of maternal care behavior that starts directly after birth and continues until weaning. These early somatosensory experiences have a strong relation to and effect on the emotional reactivity to novelty and offspring stress response later in life [171, 176, 183]. Offspring of mothers displaying disruptions in their care behavior, present with increased fearfulness compared to offspring receiving adequate or high maternal care [191, 192]. This augmented anxiety is accompanied by decreased protein and mRNA levels of glucocorticoid receptor (GR) expression within the hippocampal brain region, as well as by an impaired glucocorticoid feedback sensitivity [193]. Alterations of the GR promoter could be directly correlated to a modulation in the reactivity of the hypothalamic–pituitary–adrenal (HPA) axis [191, 192, 194]. Interestingly, cross-fostering of affected offspring to mothers displaying normal maternal care behavior reversed the behavioral effects, as well as the DNA methylation of the GR promoter, indicating the importance of postnatal care and the adverse effects of disruptions during this sensitive period [155, 194].

Furthermore, poor maternal care during the postnatal period was also associated with an adverse effect on the spatial learning and memory ability of offspring, which endured even into later phases of aging [195]. Interestingly, cross-fostering of offspring born to mothers displaying disturbed maternal care behavior, but reared by mothers showing increased maternal care, counteracted this effect [195]. These cross-fostering studies provide evidence for a direct relationship between parental behavior and offspring development. They further suggest that adverse prenatal effects may be reversed by an adequate postnatal environment, and, along this line, a poor postnatal environment may further enhance adverse prenatal influences.

Although epidemiological studies have repeatedly provided strong evidence for an association between maternal infection during pregnancy and neuropsychiatric disorders later in life in the newborn [14, 61]. On the contrary, the effect of gestational infection on the mother’s brain and possible consequences for the bonding and interaction between the mother and her newborn, remains largely unknown. This is surprising, given that the vulnerability of the female brain during the pregnancy and the perinatal period, when structural and functional rearrangements are known to occur [13, 196, 197] is well known. This is also reflected in specific psychiatric pathologies that emerge during this time window, such as postpartum depression (PPD), which severely compromises the mother’s wellbeing and her ability to relate to, and care for her newborn. While the underlying cause is not completely understood and needs further investigation, PPD has been associated with immune dysregulation, as high levels of circulating cytokines have been reported in affected women [198, 199]. Although currently still circumstantial, these observations suggest a possible link between MIA and alterations in the maternal brain, which can then result in the disruption of postnatal maternal care [196]. The relevance of postnatal care behavior and dysfunction thereof, as a possible “second hit” to an immunogenic insult during the prenatal period is, however, only beginning to be explored [74, 99, 196].

### Adaptations of the maternal brain during pregnancy and the impact of MIA

Pregnancy is accompanied by drastic physiological adaptations of the mother, including changes in the cardiovascular system [6, 200], the respiratory system [201, 202], the endocrine system [203–206], as well as neural alterations [13, 207]. Changes in women’s brain are necessary to allow the mother to form attachments with the baby, appropriately respond to their infant’s needs or detect environmental threats.

The endocrine system plays an important role during pregnancy, parturition and the postnatal period, and allows the mediation of neurological adaptations and behavioral changes [208]. During the prenatal period, progesterone levels increase enabling the implantation of the egg, as well as the maintenance of the pregnancy by inhibiting contractions and thereby preventing preterm labor [209, 210]. Furthermore, progesterone has an suppressive effect on the innate immune response [211, 212], which is important, as during pregnancy the maternal immune system has to tolerate paternal alloantigens expressed in the fetal, and also in placental tissues [213]. Regarding neural adaptions, progesterone also influences hippocampal dendritic spines [214] and acts protectively in the maternal brain [215].

The formation and density of dendritic spines in hippocampal neurons [167, 214, 216–219], as well as neurogenesis in the dentate gyrus [220], and long-term potentiation [221], are further molded by estradiol. Increased levels of estradiol are known to specifically affect brain areas directly related to maternal care behavior [222], such as the medial preoptic area (mPOA) [223], amygdala [224], hypothalamus [225, 226], olfactory system [227, 228], parietal, and prefrontal cortex [168].

The onset of parturition and initiation of maternal care is further supported by the release of oxytocin [229], which directly affects cell proliferation in the dentate gyrus [156, 230] and hippocampal long-term potentiation [231] in the female brain. Lastly, the hormone prolactin, which is secreted during the beginning of pregnancy and after parturition, mediates hippocampal neurogenesis [232, 233] and neuroprotection [234, 235] in the maternal brain. The precise timing of hormone exposure is fundamental for allowing a healthy pregnancy and adequate offspring care and is not yet well understood. Adverse events experienced by a pregnant female can not only harm the fetus directly, but also disturb maternal health and maternal care post-partum, thereby constituting a negative influence on offspring development postnatally [179].

We are only beginning to understand the dire consequences of immune stimulation on the female brain during pregnancy, a period of high malleability and augmented sensitivity of the female brain. This aspect has so far also remained largely unexplored in MIA animal models. We have recently provided first evidence that Poly (I:C) administration substantially impacts the central neural circuits mediating maternal care behavior in the female brain to fundamentally disrupt mother-infant interactions postpartum [196]. For this study we have used the most commonly used MIA Poly (I:C) protocol based upon the application of Poly (I:C) at gestational day 12.5 (20 mg/kg, i.p.) which has not increased the number of pregnancies resulting in abortion or absorption of the fetuses (*unpublished data*). These observations complement earlier results that showed altered maternal care behavior in MIA dams [96, 99]. Importantly, these studies have shown that the adverse consequences of the immunogenic stressor during pregnancy on postpartum care are not limited to the female experiencing the immune activation herself, but can be passed on her progeny [96, 99]. As such, these reports are in-line with the transgenerational transmission of MIA effects on offspring brain and behavior [61, 69, 116, 132], with the underlying mechanisms only beginning to be unraveled and likely involving changes in the epigenetic signatures [61, 69].

Jointly these data suggest always considering the variable “maternal care” in preclinical models aimed at studying the effects of perinatal risk factors.

The MIA mouse model holds significant translational value for understanding the impact of gestational infection on offspring neurodevelopmental disorders [105]. It provides a robust model system for investigating the underlying mechanisms and exploring potential therapeutic interventions [236]. However, careful consideration should be given to the limitations of the model and the challenges of translating findings obtained in experimental animals to human conditions. Humans have a complex and extended gestational period, allowing for greater interactions between the maternal immune system and the developing fetus [237]. Additionally, human brain development is rather intricated and protracted, continuing until several years after birth, being affected by social and other environmental influences later in life [3, 158, 238]. These factors may result in differences in the manifestation and severity of neurodevelopmental disorders caused by exposure to MIA. Another limitation is the heterogeneity of neurodevelopmental disorders in humans. The MIA mouse model, like any animal model, provides a reductionist representation of the complexity of human conditions to allow for experimental testing of specific hypotheses but cannot fully capture the diversity of clinical presentations and underlying diverse etiologies seen in patients. Integrating findings from animal models with clinical studies in humans will help establishing a more comprehensive understanding of the role of MIA in neurodevelopmental disorders [75].

Where, when, and how the modulatory impact of maternal care interconnects with the consequences of the immunogenic stimulus itself, remains an important topic for future investigations. We have found that neurons of the hypothalamic mPOA express receptors for cytokines induced as a consequence of Poly (I:C) stimulation and in the course of viral and bacterial infections, such as *Ifnar1* and *Ifnar2.* Additionally, mPOA neurons also express *tlr3* [196]. Thus, without further experimental data currently available, it can only be speculated whether the adverse effect of gestational infection on the maternal brain occurs directly through the contact with the pathogen, or indirectly as a result of downstream activation and release of cytokines by non-neuronal cells, including microglia or astrocytes.

Similarly, the molecular mediators, and functional interfaces at which the influences of the prenatal and postnatal environment converge to give rise to the complexity of deviations in neural development and the wide spectrum of resulting phenotypes, remains to be explored.

## Concluding remarks

The complexity of the mechanisms mediating the consequences of activation of the maternal immune system during pregnancy regulates the outcomes for offspring neurodevelopment on a wide spectrum from health to disease. The intricate interrelationship between factors of the prenatal and postnatal environments and their respective sensitivities to further external disruption, may shape an individual’s susceptibility and resilience to determine the risk for a particular pathology later in life.

## Future directions

The direct impact of immune activation on the female brain during the highly sensitive period of pregnancy shall be a topic for future research in the field. It is important to advance the understanding of the underlying cellular and molecular principles, shaping plastic alterations of neuroarchitecture and functional neurocircuitries. An aspect to consider in this context, is also the timing of the immune activation and how it relates to the neural rearrangements during pregnancy. Here it will be pivotal to determine the translational implications of the data obtained in the MIA mouse model to the human situation, under consideration of the differences in terms of the gestational timelines, and corresponding maternal and fetal developments.

Exploring how MIA impinges on the mother´s brain will be important and relevant for improving maternal health and well-being postpartum and for optimal development of the offspring.

## Data Availability

Not applicable.
